# Characterization and Genomic Analysis of ssDNA Vibriophage vB_VpaM_PG19 within *Microviridae*, Representing a Novel Viral Genus

**DOI:** 10.1128/spectrum.00585-22

**Published:** 2022-07-06

**Authors:** Ruizhe Guo, Kaiyang Zheng, Lin Luo, Yundan Liu, Hongbing Shao, Cui Guo, Hui He, Hualong Wang, Yeong Yik Sung, Wen Jye Mok, Li Lian Wong, Yu-Zhong Zhang, Yantao Liang, Andrew McMinn, Min Wang

**Affiliations:** a College of Marine Life Sciences, Frontiers Science Center for Deep Ocean Multispheres and Earth System, Institute of Evolution and Marine Biodiversity, Ocean University of China, Qingdao, China; b UMT-OUC Joint Centre for Marine Studies, Qingdao, China; c Institute of Marine Biotechnology, University Malaysia Terengganu (UMT), Kuala Nerus, Malaysia; d State Key Laboratory of Microbial Technology, Marine Biotechnology Research Center, Shandong University, Qingdao, China; e Institute for Marine and Antarctic Studies, University of Tasmania, Hobart, Tasmania, Australia; f The Affiliated Hospital of Qingdao University, Qingdao, China; University of Pittsburgh School of Medicine

**Keywords:** *Vibrio*, phage vB_VpaM_PG19, *Microviridae*, genomic and phylogenetic analysis

## Abstract

Vibrio parahaemolyticus, a widespread marine bacterium, is responsible for a variety of diseases in marine organisms. Consumption of raw or undercooked seafood contaminated with V. parahaemolyticus is also known to cause acute gastroenteritis in humans. While numerous dsDNA vibriophages have been isolated so far, there have been few studies of vibriophages belonging to the ssDNA *Microviridae* family. In this study, a novel ssDNA phage, vB_VpaM_PG19 infecting V. parahaemolyticus, with a 5,572 bp ssDNA genome with a G+C content of 41.31% and encoded eight open reading frames, was isolated. Genome-wide phylogenetic analysis of the total phage isolates in the GenBank database revealed that vB_VpaM_PG19 was only related to the recently deposited vibriophage vB_VpP_WS1. The genome-wide average nucleotide homology of the two phages was 89.67%. The phylogenetic tree and network analysis showed that vB_VpaM_PG19 was different from other members of the *Microviridae* family and might represent a novel viral genus, together with vibriophage vB_VpP_WS1, named *Vimicrovirus*. One-step growth curves showed that vB_VpaM_PG19 has a short incubation period, suggesting its potential as an antimicrobial agent for pathogenic V. parahaemolyticus.

**IMPORTANCE** Vibriophage vB_VpaM_PG19 was distant from other isolated microviruses in the phylogenetic tree and network analysis and represents a novel microviral genus, named *Vimicrovirus*. Our report describes the genomic and phylogenetic features of vB_VpaM_PG19 and provides a potential antimicrobial candidate for pathogenic V. parahaemolyticus.

## INTRODUCTION

Vibrio parahaemolyticus is a short, rod-shaped halophilic Gram-negative bacterium that is widely distributed in seas and estuaries. It is known to infect shrimp, resulting in acute hepatopancreatic necrosis disease (AHPND), which can lead to the early death of shrimp, with consequent huge economic losses (1). As a major foodborne pathogen, V. parahaemolyticus is also considered to be an important human health problem. When raw or undercooked seafood is eaten, it can cause gastroenteritis and sepsis (2). Recently, a rise in ocean temperature has led to an increase in vibriosis outbreaks, which suggests that *Vibrio* infections will increase in a future warmer ocean (3–5). This emphasizes the importance of prevention and control of *Vibrio*-associated diseases (6–8). In recent decades, with the widespread use of antibiotics, antibiotic resistance has increased significantly, leading to the emergence of multidrug-resistant bacteria in aquaculture, especially as human pathogens (9). Therefore, there is an urgent need to find alternative strategies to prevent and control antibiotic-resistant pathogenic bacteria, such as V. parahaemolyticus.

Phages are viruses that specifically infect bacteria. They are the most abundant and diverse “biological entities” on earth (10–12). Phage genomes contain many new genes with unknown functions and so may be one of the largest unexplored gene pools (13). Unlike antibiotics, lytic phages can control and kill bacteria, can undergo modification during the coevolution process between phage and bacteria to continue to infect and lyse them, and can also adapt to antibiotic-resistant strains (14). With the inevitable emergence of further multidrug-resistant bacteria and the slowdown in the discovery of new antibiotics, phage therapy is receiving continuing attention (13). There have already been several studies on the identification and animal application of vibriophages; these include phages VP-1, VP-2, VP-3, PVS-1, PVS-2, PVS-3, vB_VpaP_VP-ABTNL-1, and vB_VpaS_VP-ABTNL-2 (13, 15). Phage therapy is considered to be an environmentally friendly method with great potential to reduce and control antibiotic-resistant V. parahaemolyticus.

However, most isolated vibriophages have been double-stranded DNA (dsDNA) phages, and there have been few single-stranded DNA (ssDNA) phage isolates. The few that have been isolated are mostly in the *Inoviridae* family (16). *Microviridae* is a family of icosahedral lytic bacteriophages with circular single-stranded DNA genomes. Our understanding of lytic ssDNA phages is mainly derived from this family, which contains two major subfamilies, i.e., *Bullavirinae* and *Gokushovirinae* (recognized by the International Committee on Taxonomy of Viruses [ICTV; 17]). Here, for the first time, we report on the morphological, genomic, and phylogenetic characteristics of the vibriophage isolated from V. parahaemolyticus and named vB_VpaM_PG19, which represents a novel viral genus. This study has expanded our understanding of ssDNA phages within *Microviridae* and provided a new potential antimicrobial candidate for pathogenic V. parahaemolyticus.

## RESULTS AND DISCUSSION

### Phage morphology, host range, growth curve, and pH sensitivity.

**FIG 1:**
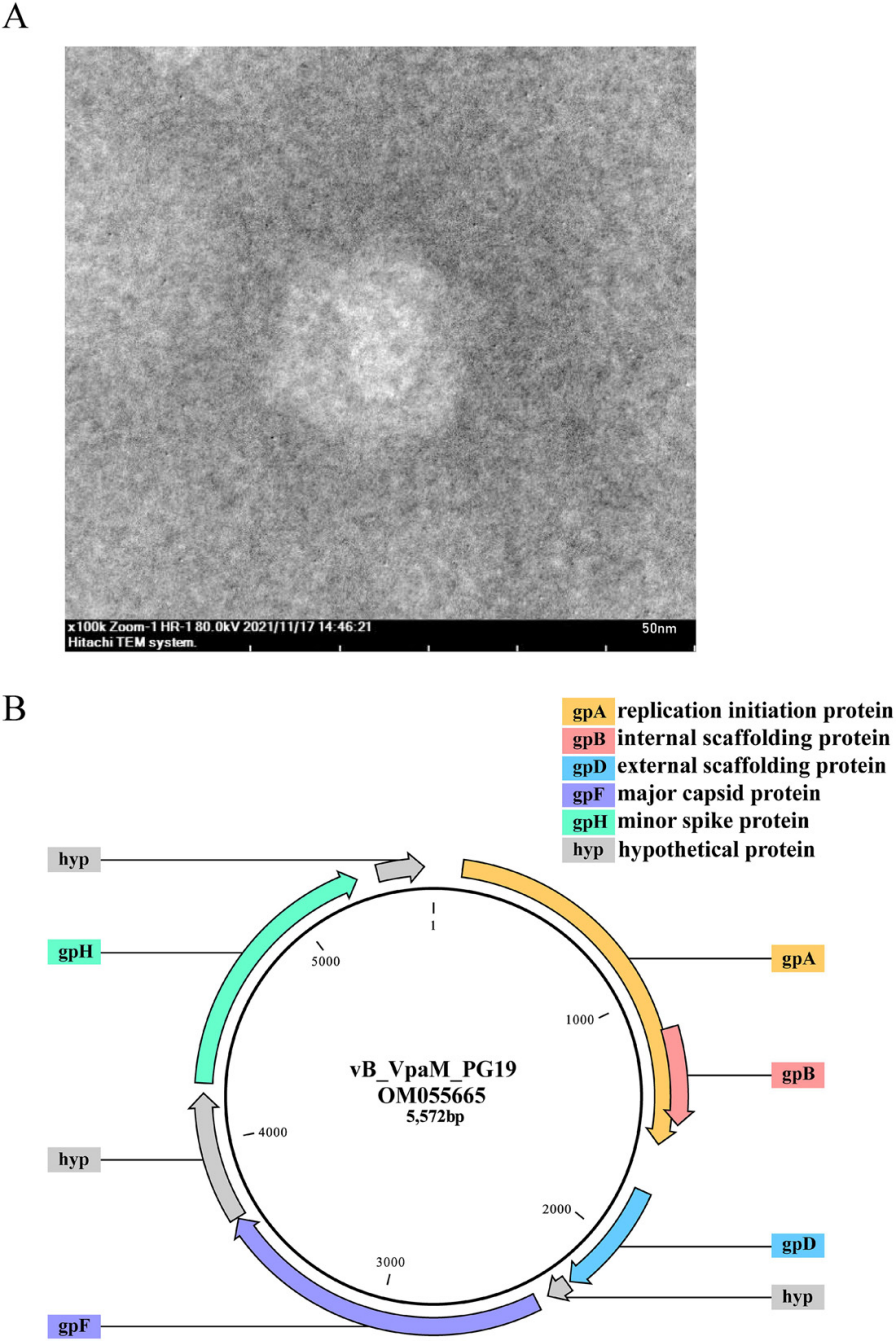
(A) Transmission electron microscopy (TEM) morphology of vibriophage vB_VpaM_PG19. Phages were negatively stained with potassium phosphotungstate. Scale bar, 50 nm. (B) Genome map of vibriophage vB_VpaM_PG19. Putative functional categories were defined according to annotation and are represented by different colors. The length of each arrow represents the length of each gene.

**FIG 2:**
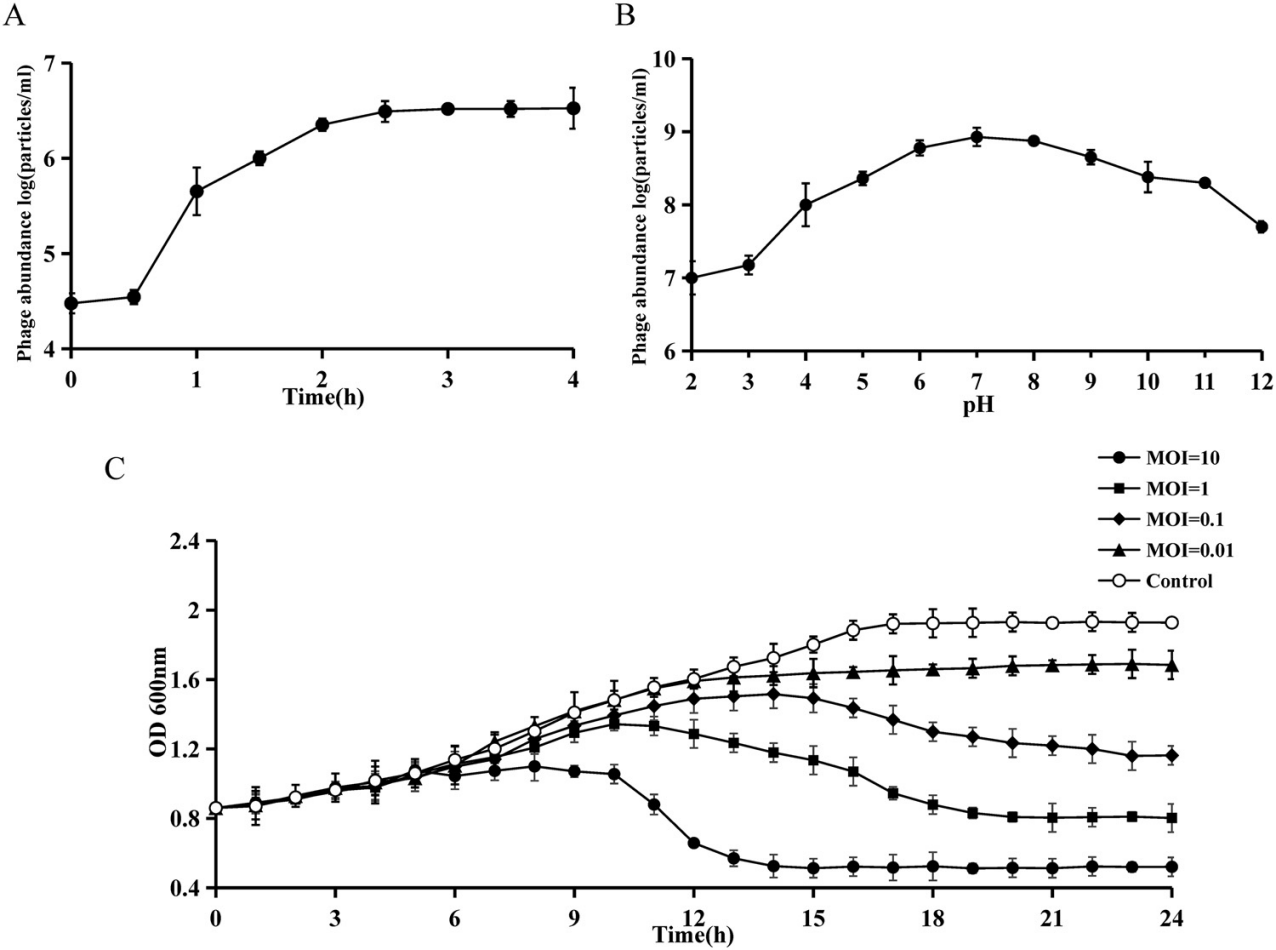
Biological properties of vibriophage vB_VpaM_PG19. (A) One-step growth curve of vibriophage vB_VpaM_PG19. The data shown are average values from triplicate experiments, and error bars indicate standard deviations (SDs). (B) pH stability curve of vibriophage vB_VpaM_PG19. The data shown are average values from triplicate experiments, and error bars indicate standard deviations (SDs). (C) Lytic effect of phage vB_VpaM_PG19 against Vibrio parahaemolyticus (GenBank accession NR_114630.1) *in vitro*. V. parahaemolyticus was infected by phage vB_VpaM_PG19 at MOI of 0.01, 0.1, 1, or 10 and cultured for up to 24 h. V. parahaemolyticus cultured with the same volume of phage diluent was used as a control. This experiment was repeated three times, and the data are shown as mean ± SEM.

The transmission electron microscopy (TEM) images show that vB_VpaM_PG19 has an isometric capsid with T = 1 icosahedral symmetry, with a diameter of 22 ± 0.5 nm and without a tail (Fig. 1A), conforming to the reported size and morphologies of phages within the *Microviridae* family. The host cross-infection experiment showed that phage vB_VpaM_PG19 has a narrow host range. Of the 13 strains tested, it was found to only infect three strains of Vibrio parahaemolyticus ATCC 17802, Vibrio tasmaniensis LMG 21574, and Vibrio hangzhouensis CN83 (Table 1). A one-step growth curve showed that the latent period of phage vB_VpaM_PG19 was 30 min and the burst size was approximately 11 virions per cell (Fig. 2A). Phage vB_VpaM_PG19 was stable from pH 2 to 12 in pH sensitivity experiments. However, the activity under acidic conditions was weaker than that under alkaline conditions. This showed that vB_VpaM_PG19 can survive in a range of acid-base conditions but had stronger adaptability to an alkaline environment (Fig. 2B). To determine the bacteriolytic activity of phage vB_VpaM_PG19, V. parahaemolyticus was infected with phage vB_VpaM_PG19 at MOI of 0.01, 0.1, 1, or 10 at 25°C. Culture samples were collected at 1-h intervals for 24 h, and bacterial growth was measured based on OD600, suggesting that the growth of the V. parahaemolyticus was inhibited when cocultured with a phage in a concentration-dependent manner, with OD600 values declining more quickly at MOI 10 than at MOI 0.01, 0.1, or 1 (Fig. 2C).

**TABLE 1:** Host range analysis of vibriophage vB_VpaM_PG19

Strain	Susceptibility
Vibrio parahaemolyticus ATCC 17802	+
Vibrio neocaledonicus NC470	–
Vibrio tasmaniensis LMG 21574	+
Vibrio kanaloae LMG 20539	–
Vibrio natriegens DSM 759	–
Vibrio plantisponsor MSSRF60	–
Vibrio hangzhouensis CN83	+
Vibrio atlanticus Vb 11.11	–
Vibrio splendidus ATCC 33125	–
Vibrio algicola SM1977	–
Vibrio algivorus SA2	–
Vibrio rumoiensis S-1	–
Vibrio sagamiensis LC2-047	–

### Overall genome features of vB_VpaM_PG19.

Phage vB_VpaM_PG19 contained a 5,572 bp ssDNA genome with a G+C content of 41.31%. No tRNA genes were predicted (Fig. 1B). The ORFs of vB_VpaM_PG19 were predicted by multiple methods, including GeneMarkS, GeneMark.hmm, RAST, and ORFfinder. A total of eight ORFs were predicted (Table 2). The ORFs of vB_VpaM_PG19 were identified by BLASTp and Pfam search, and a total of five ORFs were predicted, including gpA (ORF1), gpB (ORF2), gpD (ORF3), gpF (ORF5), and gpH (ORF7). GpA encodes the replication initiation protein, which is homologous to gpA in phiX174 and VP4 in Chp1. GpB is an overlapping gene and located inside gpA, encoding the internal scaffolding protein, and there are consistent functions for gpB in phiX174 and VP3 in Chp1. GpD encodes the external scaffolding protein, homologous to gpD in phiX174. GpF encodes the major capsid protein, which has the same function as gpF in phiX174 and VP1 in Chp1. GpH encodes the pilot protein, which is functionally parallel to gpH of phiX174 and VP2 of Chp1 (18–20) (Table 3).

**TABLE 2:** Genomic annotation of the open reading frames (ORFs) of vibriophage vB_VpaM_PG19 and conserved domains detected

ID no.	Start	Stop	Strand	Annotated function	BLASTP result			Pfam result		
					Accession	E value	Per identity	Bit score	E value	Accession
ORF1	114	1580	+	Replication initiation protein	AXF53132.1	3.00E-111	39.64%	66.4	2.50E-18	PF05840
ORF2	1142	1498	+	Internal scaffolding protein	AXF53131.1	7.00E-08	34.17%	37.7	2.40E-09	PF02304
ORF3	1771	2223	+	External scaffolding protein	WP_031618335.1	8.00E-51	52.82%	170	2.90E-50	PF02925
ORF4	2236	2325	+	Unknown						
ORF5	2373	3683	+	Major capsid protein	AAZ49266.1	4.00E-125	45.98%	148.5	2.80E-43	PF02305
ORF6	3689	4192	+	Unknown						
ORF7	4232	5272	+	Pilot protein				22.9	5.00E-05	PF04687
ORF8	5355	5537	+	Unknown						

**TABLE 3:** Proteins found in representative members of the family *Microviridae*^*a*^

PG19 proteins	phiX174 proteins	Chp1 proteins	Protein function
gpA	gpA	Vp4	Replication initiation protein
Absent	gpA	Unknown	Unknown
gpB	gpB	Vp3	Internal scaffolding protein
Absent	gpC	Vp5(?)	ssDNA synthesis & inhibitor of dsDNA
gpD	gpD	Absent	External scaffolding protein
Absent	gpE	Unknown	Lysis protein
gpF	gpF	Vp1	Major caspid protein
Unknown	gpG	Absent	Major spike protein
gpH	gpH	Vp2	Minor spike protein
Unknown	gpJ	Vp8(?)	DNA binding protein
Absent	gpK	Unknown	Unknown
Absent	absent	Vp6, Vp7	ORF coding capacity and/or protein function unknown

a “Absent” indicates that no homologue exists. “Unknown” indicates that the protein has not been identified or the identity of the homologue, if it exists, is not readily apparent. (?) indicates a hypothesized function.

### Phages without major spike proteins but with external scaffolding proteins.

According to the taxonomy releases of viruses by the International Committee on Taxonomy of Viruses (ICTV) in 2020, *Microviridae* contains two major subfamilies: *Bullavirinae* and *Gokushovirinae*. These subfamilies differ from their hosts’ genome structure and virion composition. Members of *Gokushoviridae* were originally thought to occupy a unique ecological niche, infecting obligate intracellular bacteria. However, genome analysis has shown that this population also infects free-living hosts (21). Members of the *Bullavirinae* subfamily generally infect Enterobacteria (20, 22). In recent years, *Microviridae* phages infecting other bacterial groups have also been discovered, suggesting that ssDNA bacteriophages should be more diverse than previously thought (23–28). The main structural basis for distinguishing the two subfamilies is that the icosahedral capsid of *Bullavirinae* has 12 spikes on the 5-fold vertices, which are constructed from the major spike protein (gpG in phiX174) (29), whereas *Gokushovirinae* has no spikes on the 5-fold vertices. Instead, there are “mushroom-like” protrusions at the 3-fold vertices of the icosahedral capsid (20), which are formed by an insertion loop encoded within the hypervariable region of the major capsid protein (19, 26, 30, 31). In addition, in the *Gokushovirinae* subfamily, the assistance of external scaffolding proteins is not necessary for capsid assembly (32–34), so the phages in this subfamily lack the external scaffolding gene (gpD in phiX174). The genome size of phage in the *Gokushovirinae* subfamily is about 20% smaller than that in the *Bullavirinae* subfamily due to the lack of gpG and gpD homologs.

**FIG 3:**
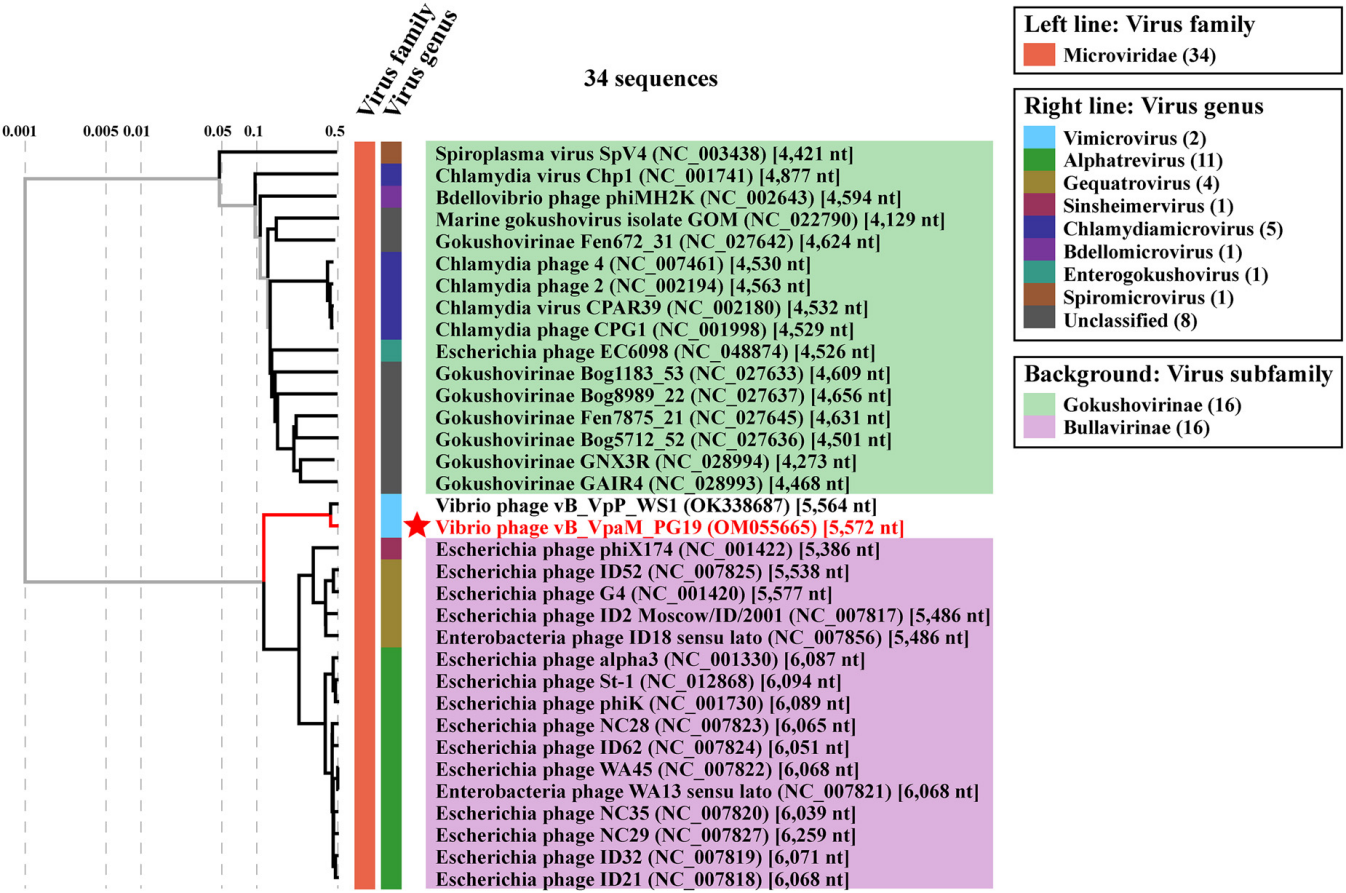
Proteomic tree of 32 classified *Bullavirinae* and *Gokushovirinae* subfamily reference sequences from the NCBI virus database and 2 queries (vB_VpaM_PG19 and vB_VpP_WS1), constructed by ViPTree. The colored bands represent the virus families (left band) and virus genera (right band). The different colors of the background represent the virus subfamilies. This tree is calculated by BIONJ according to the genome distance matrix and takes the midpoint as the root.

**FIG 4:**
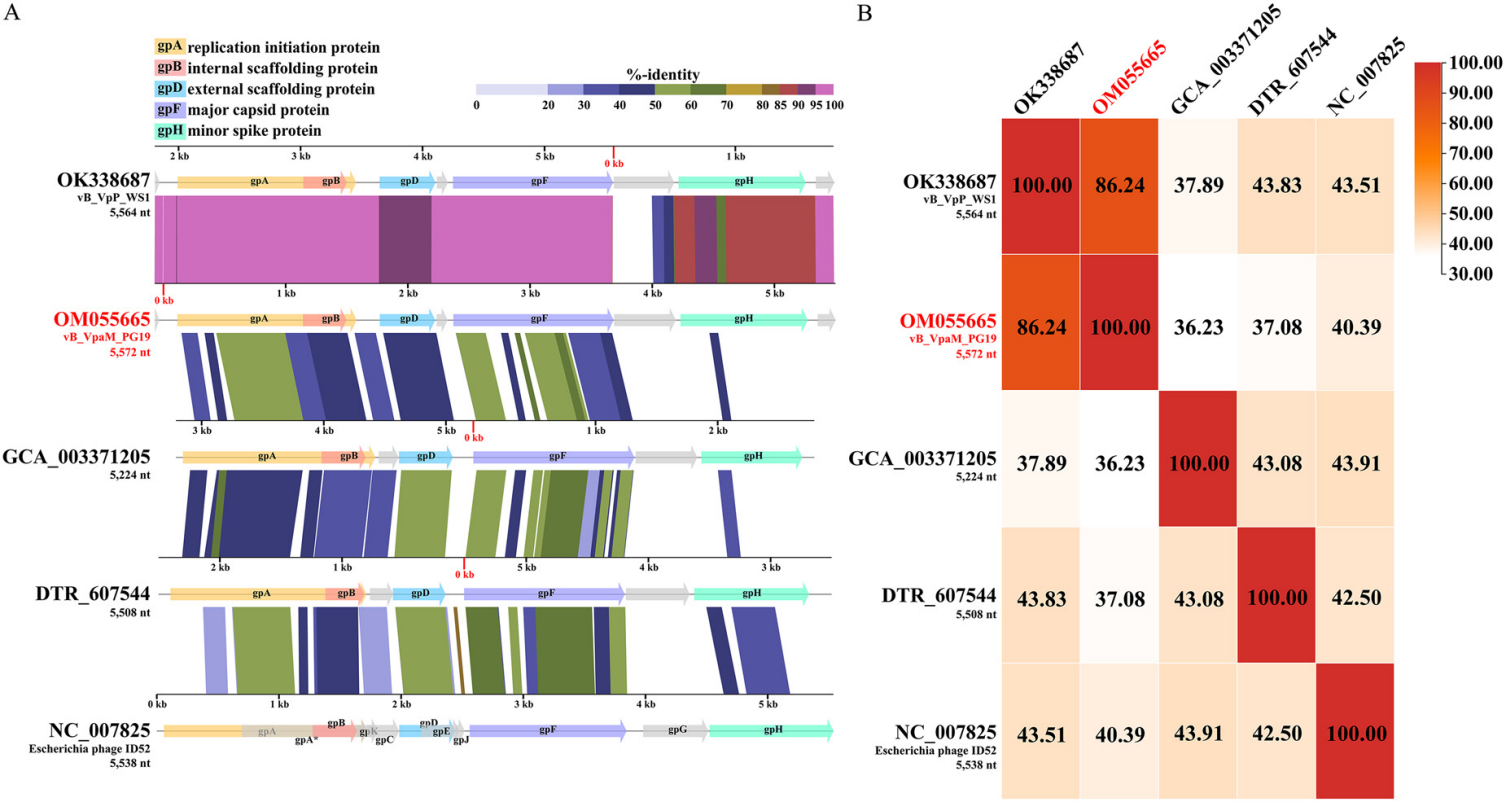
(A) Genomic comparisons between vB_VpaM_PG19, vB_VpP_WS1, GCA_003371205, DTR_607544, and Escherichia phage ID52. The predicted functions of proteins are indicated by different colors of arrows representing genes. The shading below each genome indicates sequence similarities between the genomes, with different colors representing the levels of similarity. (B) The heat map shows AAI among vB_VpaM_PG19, vB_VpP_WS1, two contigs (GCA_003371205 and DTR_607544) from the IMG/VR database that were closest to vB_VpaM_PG19 in the result of the multigene maximum-likelihood phylogenetic tree, and a typical microvirus Escherichia phage ID52 that has the highest AAI with vB_VpaM_PG19 among the NCBI virus reference sequences. The ratio of AAI was based on an AAI calculator.

To determine which subfamily is more closely related to vB_VpaM_PG19, a proteomic tree based on the similarities of the whole genome was generated using ViPTree (https://www.genome.jp/viptree/) (35) (Fig. 3). The results showed that the position of vB_VpaM_PG19 in the phylogenetic tree was closer to *Bullavirinae*. However, vB_VpaM_PG19 originated from the tree root and formed a separate clade, and although a homolog of gpD was present, a homolog of gpG was not found in the genome of phage vB_VpaM_PG19 (Fig. 4A). This means that vB_VpaM_PG19 cannot be classified into any subfamily within *Microviridae* by current definitions. This demonstrates the possibility that vB_VpaM_PG19 may represent a novel *Microviridae* cluster.

### Phage vB_VpaM_PG19 represents a new microviral genus.

The result of average nucleotide identity (ANI) between vB_VpaM_PG19 and vB_VpP_WS1, calculated by OrthoANI, was 89.67%. However, OrthoANI is not functional when the ANI is less than 70%. Therefore, the sequence similarity based on average amino acid identity (AAI) was confirmed. The all-versus-all AAI analysis among vB_VpaM_PG19, vB_VpP_WS1, ID52, and two contigs from the IMG/VR database was calculated by the AAI calculator (36). vB_VpaM_PG19 had the highest similarity with vB_VpP_WS1, with an AAI of 86.24%; the AAIs of other sequences were less than 50% (Fig. 4B).

The Bacterial and Archaeal Viruses Subcommittee (BAVS) of the International Committee on the Taxonomy of Viruses (ICTV) considers phages sharing ≥50% ANI as members of the same genus (37). The high ANI (89.67%) between vB_VpaM_PG19 and vB_VpP_WS1 was thus in accord with the threshold for the formation of a new genus, which was further supported by the results of AAI analysis (Fig. 4B).

**FIG 5:**
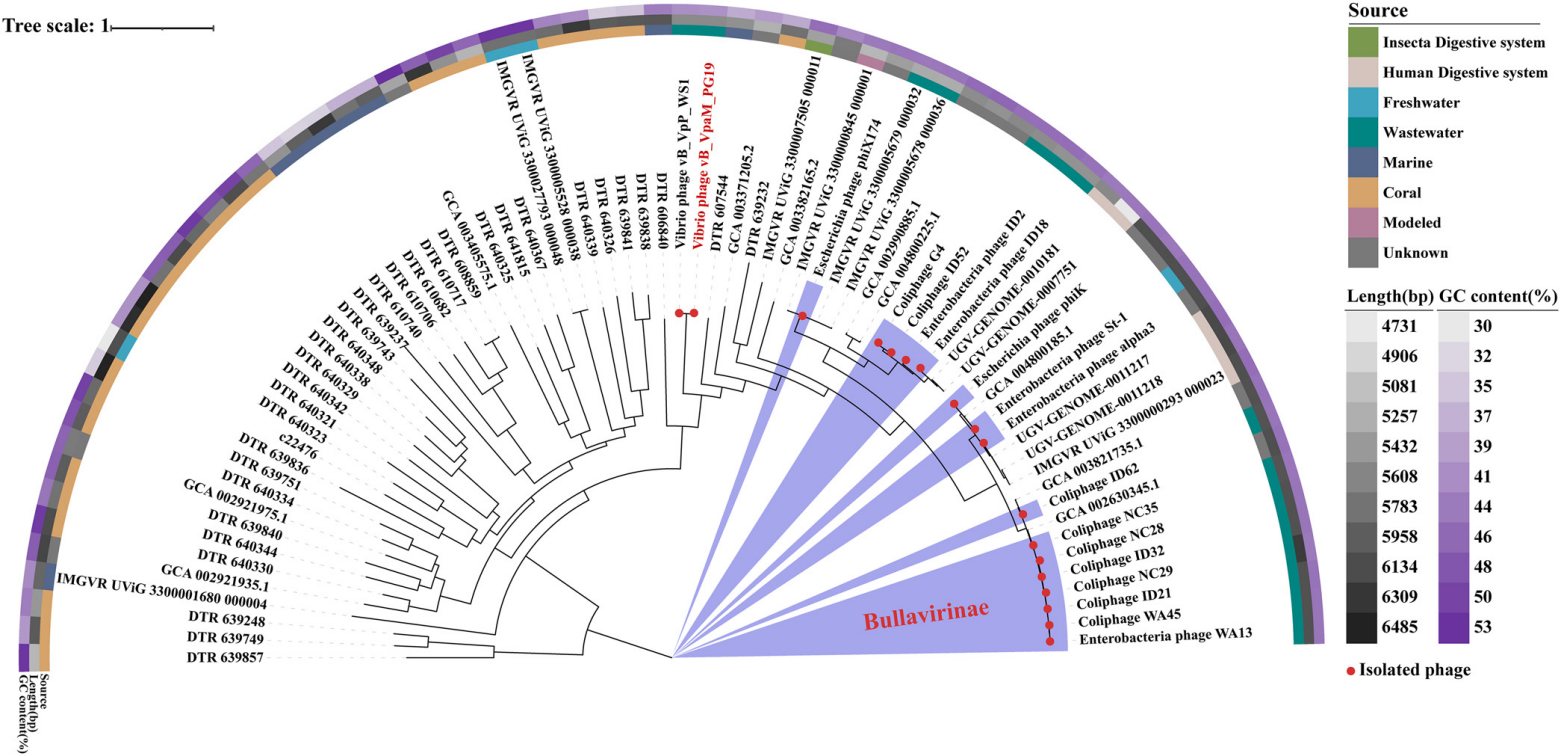
Three genes (replication initiation protein, external scaffolding protein, and major capsid protein) from 73 related sequences were connected and aligned to build a multigene maximum-likelihood phylogenetic tree using IQtree. The colored rings represent the source of sequences (inner ring), length (middle ring), and GC content (outer ring). The branches of red dots represent isolated phages, while the branches of unmarked dots represent contigs from the IMG/VR database.

**FIG 6:**
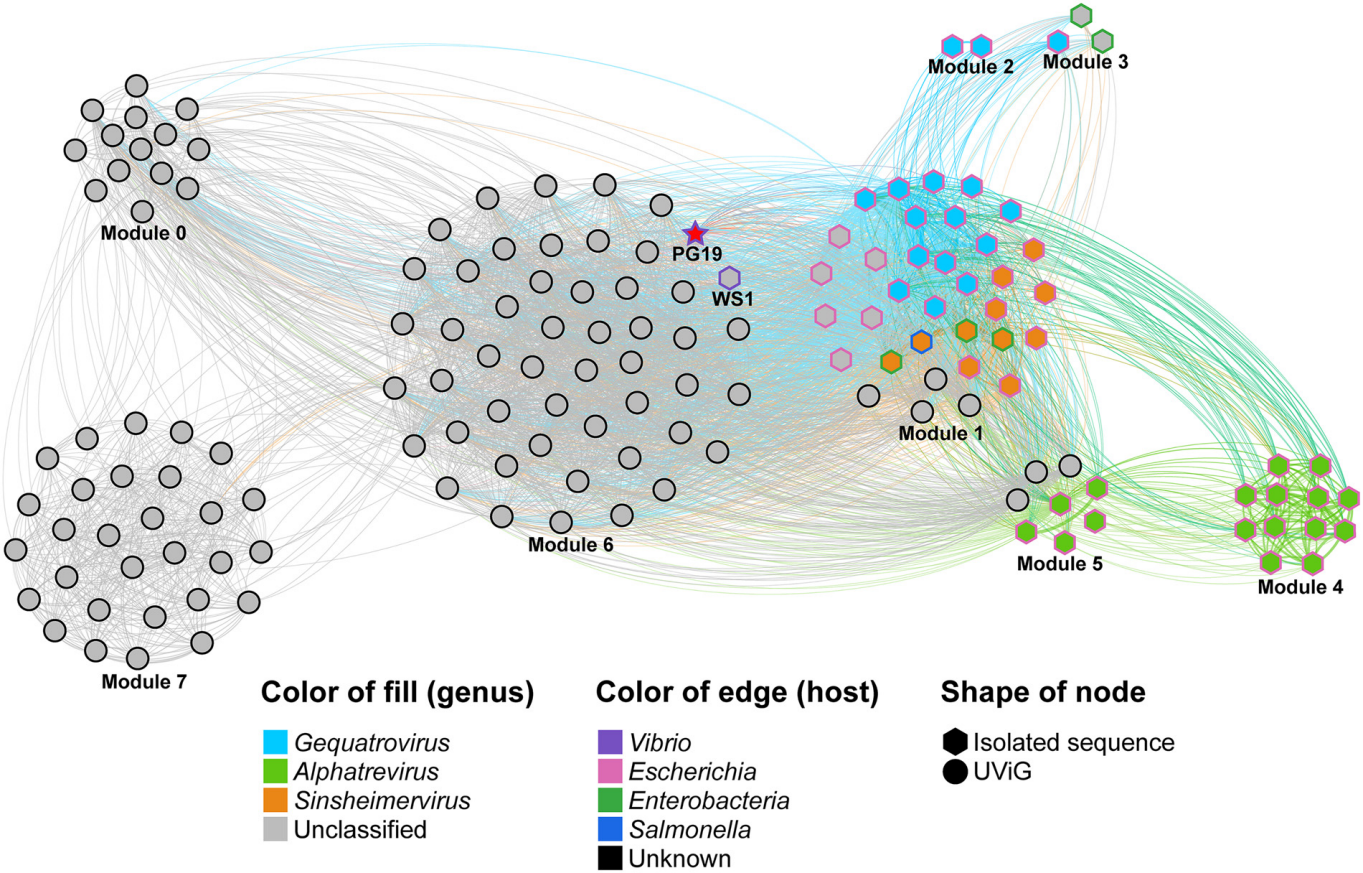
Gene content-based viral network among vB_VpaM_PG19 and vB_VpaM_PG19-associated genomes from the NCBI virus database and IMG/VR database. The nodes represent the viral genomic sequences. The edges represent the hosts. The isolated viral sequences are indicated by regular hexagons, and uncultured viral genomes (UviGs) from IMG/VR are indicated by filled circles. Among those, the star represents vibriophage vB_VpaM_PG19. Viral genomes that belong to different genera are indicated by different colors. Vibriophage vB_VpaM_PG19 is shown in red.

To further understand the phylogenetic relationship between vB_VpaM_PG19, vB_VpP_WS1, and other isolated phages, 32 classified *Bullavirinae* and *Gokushovirinae* subfamily reference sequences from the NCBI virus database were selected, with vB_VpaM_PG19 and vB_VpP_WS1 together, to establish the whole-genome phylogenetic tree. Among them, vB_VpaM_PG19 and vB_VpP_WS1 originated from the tree root and formed a separate clade (Fig. 3). The multi-gene maximum-likelihood phylogenetic tree (Fig. 5) was generated after picking sequences in IMG/VR that were similar to vB_VpaM_PG19. It can be seen that vB_VpaM_PG19 and vB_VpP_WS1 were grouped together and formed a unique viral cluster. In addition, a gene content-based viral network analysis among vB_VpaM_PG19 and other related viral genomes was performed, including viral genomes from the NCBI virus database and the IMG/VR database. The result showed that vB_VpaM_PG19 did not belong to any of the currently classified genera and was distant from known microvirus genomes (Fig. 6). All sequences in the same module as vB_VpaM_PG19 are UViGs except vB_VpP_WS1.

**FIG 7:**
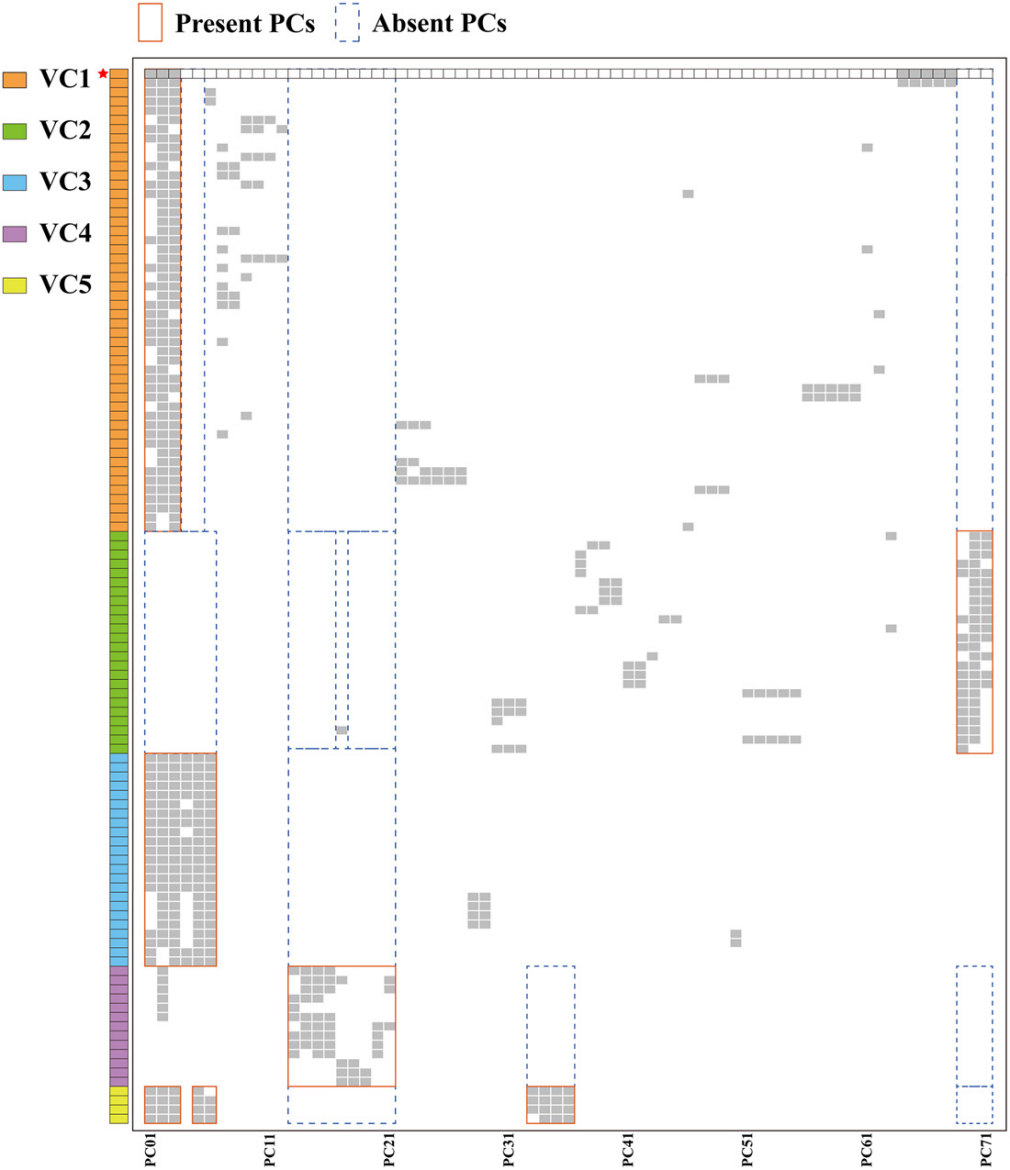
Protein clusters analysis between vB_VpaM_PG19 and vB_VpaM_PG19-associated genomes from the NCBI virus database and IMG/VR data set. Blocks on the left represent different genomes, and different colors represent different viral clusters (VCs). The solid red border indicates protein clusters (PCs) presented, and the dotted blue border indicates PCs absented. vB_VpaM_PG19 is labeled as a red star.

The presence and absence of specific protein sequences could complement the basis for the classification of the *Microviridae* family and help find taxonomic status for microviruses that have not yet been classified (23). Hence, a protein sharing analysis between vB_VpaM_PG19 and associated genomes was carried out, after the sequences with a genome size shorter than 4,000 bp were screened out based on the genome length of reference sequences labeled *Microviridae* in the NCBI data set (Fig. 7). The heatmap showed that vB_VpaM_PG19 and vB_VpaM_PG19-associated genomes were divided into five viral clusters (VCs), with different protein clusters (PCs) in each VC. The vB_VpaM_PG19 (labeled as red star) was clustered into VC1. The sequences of VC1 contained at least two proteins of PC1, PC2, and PC3, but did not contain PC4, PC5, and PC13-PC21. The sequences of VC2 contained at least two proteins of PC69, PC70, and PC71 but did not contain PC1-PC6, PC13-PC16, and PC18-PC21. The sequences of VC3 contained at least four proteins of PV1-PC6 but did not contain PC13-PC21. The sequences of VC4 contained at least one protein of PC13-PC21 but did not contain PC33-PC36. The sequences of VC5 contained at least eight proteins from PC1-PC3, PC5, PC6, and PC33-PC36 but did not contain PC13-PC21. In addition, both vB_VpaM_PG19 and vB_VpP_WS1 (second row) contained PC64-PC68, and these five proteins were not detected in other sequences, reflecting the specificity of these two phage genomes. As the only two isolated microviruses infecting genus *Vibrio* and classified into a novel viral cluster, we propose that vB_VpaM_PG19 and vB_VpP_WS1 represent a novel viral genus, named *Vimicrovirus*, within the *Microviridae*.

### Conclusion.

*Vibrio* is an important marine pathogenic bacterial genus with a very special metabolic capacity and ecological niche. Consequently, its phage will inevitably affect its abundance, community structure, and metabolic capacity. vB_VpaM_PG19 is a rare ssDNA microvirus infecting *Vibrio* and represents a novel microviral cluster, together with vB_VpP_WS, distant from the two subfamilies within *Microviridae*. vB_VpaM_PG19 and vB_VpP_WS1 have low similarity to existing isolated phages and contigs from the metagenomic database. Thus, we propose vB_VpaM_PG19 as a representative member of a new microviral genus, named *Vimicrovirus*, within the *Microviridae* family to reconcile this cluster. Our report provides an in-depth analysis of phages of the *Vimicrovirus* genus characterized at the genomic, phylogenetic, and ecological levels and provides a potential antimicrobial candidate for pathogenic V. parahaemolyticus.

## MATERIALS AND METHODS

### Sampling.

The sample used here was collected from the sewage outflow of the Qingdao seafood market (36°N,120°/E) in April 2021 and stored at 4°C.

### Bacterial strain.

The bacterial strain was provided by Yuzhong Zhang’s laboratory at Shandong University and cultured in 2216E media at 25°C. The molecular identification of the isolate was obtained by 16S rRNA gene sequence analysis, and the homology of the 16S rRNA gene sequence was then studied by BLAST search (38). The 16SrRNA of the isolated host strain was most similar to Vibrio parahaemolyticus ATCC 17802, which was a pathogenic strain (39) (percent of identity at 99.46%). Stock cultures were stored in 2216E broth supplemented with 30% glycerol at −80°C.

### Isolation and concentration of the phage.

The sewage sample was filtered through a 0.22 μm Polyethersulfone (PES) Millipore filter, to remove the bacteria and phytoplankton. The phages in the sample were then separated by the double agar layer method. In summary, 200 μL of sewage sample filtrate and 200 μL of logarithmic growth phase bacterial were mixed into a suspension in a cryopreservation tube and left to stand at 25°C for 30 min. The mixture was then injected into 4.5 mL of semisolid medium melted at 50°C and poured onto the surface of the solid medium. After culturing the agar plate in an incubator at 25°C for 24 h, the formation of plaques was observed. If there was plaque on the plate, it was picked out and placed in 1 mL of SM Buffer (100 mM NaCl, 8 mM MgSO_4_·7H_2_O, 50 mM Tris-Cl, pH = 7.5), and then filtered onto a 0.22 μm PES Millipore filter. After the filtrate was gradually diluted, the above procedure was repeated. The infection step was repeated at least three times to ensure that the phage solution was completely purified. The purified phage solution was cultured to 500 mL and treated overnight with 10% (wt/vol) PEG6000 at 4°C to precipitate the phages. The next day, the sample was centrifuged at 4,500 *g* at 4°C for 30 min, and then the supernatant was gently poured off without disturbing the precipitate. The precipitate was resuspended in 5 mL SM Buffer. The purified phage solution was stored in SM Buffer at 4°C until processing (40).

### Transmission electron microscopy.

The concentrated and purified viral suspension in a volume of 20 μL was applied to a 200-mesh copper grid and negatively stained with 1% (wt/vol) phosphotungstic acid (pH 7.0). Their morphology was examined on a transmission electron microscopy (HT7700 Exalens, Japan) at 80 kV to identify the structural features (41). Each grid on the scale represents 50 nm.

### DNA extraction and genome sequencing.

Viral nucleic acid was extracted using an OMEGA viral DNA kit according to the manufacturer's instructions. Purified phage genomic DNA was sequenced by the Illumina NovaSeq 2 × 150 bp paired-end sequence method. Gaps between remaining contigs were closed via the Gap Closer v1.12. Open reading frames (ORFs) were analyzed by GeneMarkS (42), GeneMark.hmm (43), RAST (https://rast.nmpdr.org/rast.cgi), and ORFfinder (https://www.ncbi.nlm.nih.gov/orffinder/). Gene functions were predicted by finding homologs in the nonredundant database (http://blast.ncbi.nlm.nih.gov/) (44, 45) and searching for conserved domains in the Pfam database (http://pfam.xfam.org/).

### Homolog genomes in the IMG/VR data set.

After comparing vB_VpaM_PG19 with the isolated phages in the genome database, only one closely related microvirus was found, vibriophage vB_VpP_WS1. This had a relatively high ANI (89.67%) and average amino acid identity (AAI, 86.24%), perhaps because these are the only two isolated microviruses that infect *Vibrio*. To expand the phage vB_VpaM_PG19 group, all sequences annotated as *Microviridae* in the IMG/VR v3 database (46) were queried by tBLASTx. Homologous sequences (threshold: E value <1e-5) were sought and contig IDs mapped, finding a total of 118 sequences. Homologous sequences from the IMG/VR database, *Microviridae* family reference sequences from the NCBI virus database, and vibriophage vB_VpP_WS1 were integrated to remove redundancy, and GenemarkS was used to predict ORFs. Seventy-three sequences containing gpA (replication initiation protein), gpD (external scaffolding protein), and gpF (major capsid protein) homologous to vB_VpaM_PG19 (threshold: E value <1e-5) were screened using BLASTp, and their gpA, gpD, and gpF were connected in series to establish a multigene maximum-likelihood phylogenetic tree. The phylogenetic tree was generated using MAFFT V7 and visualized with iTOL v4 (47).

### Phylogenetic and comparative genomic analysis.

A proteomic tree based on the similarities of the whole genome was generated using ViPTree. The phylogenetic tree was generated using 32 classified *Bullavirinae* and *Gokushovirinae* subfamily reference sequences from the NCBI virus database; vB_VpaM_PG19 and vB_VpP_WS1 were used as queries to construct the whole-genome phylogenetic tree. vB_VpaM_PG19, vB_VpP_WS1, two contigs (GCA_003371205 and DTR_607544) from the IMG/VR database that were closest to vB_VpaM_PG19 in the results from the multigene maximum-likelihood phylogenetic tree, and Escherichia phage ID52, which has the highest AAI with vB_VpaM_PG19 among the NCBI virus reference sequences, were selected to perform the multigenomic alignments.

### Network analysis with microviruses from NCBI virus and IMG/VR.

Sequences from all microviruses in the NCBI virus database and all contigs annotated as *Microviridae* in the IMG/VR database were placed into a single set together with vB_VpaM_PG19. All-to-all BLASTn was performed for the 15,276 *Microviridae* genomes to remove duplications with the nondefault parameters: E value 1e-10, max_hsps 1, max_target_seqs 100,000, perc_ident 99, qcovs_per_hsp 100. In total, 3,051 duplicate genomes were found and removed from the original set, resulting in 12,225 nonredundant genome sets. The genome of vB_VpaM_PG19 was subjected to tBLASTx against the nonredundant genome set with the nondefault parameters (E value 1e-10, max_hsps 1, max_target_seqs 100,000); 87 vB_VpaM_PG19-associated microviral genomes were retrieved. vB_VpaM_PG19 and 87 vB_VpaM_PG19-associated genomes were combined and subjected to tBLASTx against the nonredundant genome set with the same parameters; 64 extra PG19 indirectly associated microviral genomes were retrieved. The intergenomic relationships were analyzed based on the set containing vB_VpaM_PG19 and 151 vB_VpaM_PG19-associated genomes. vB_VpaM_PG19 and all vB_VpaM_PG19-associated genomes were subjected to BLAST to perform all-to-all tBLASTx, with the parameters: E value 1e-10, max_target_seqs 100,000; 135,972 high-scoring pairs (hsps) were generated. The bit scores from reciprocal hit pairs were averaged to eliminate the scoring bias between queries and subjects. To eliminate the impact resulting from differing genome lengths of these microviral genomes, bit scores of hsps were summed for each query–subject pair and normalized by the total length of the two genomes. Normalized bit scores of query–subject pairs as the weight of edges were used to build the binary profile of the genomic relationship network. The layout of the network was calculated from the binary profile based on the Force Atlas algorithm. The modularization of the intergenomic relationship network was calculated with the default resolution.

### Protein clusters between vB_VpaM_PG19 and vB_VpaM_PG19-associated genomes.

vB_VpaM_PG19 and 151 vB_VpaM_PG19-associated genomes were subjected to BLAST to perform all-to-all tBLASTx with the parameters E value 1e-10, max_target_seqs 100,000; 135,972 high scored pairs were generated. The bit scores from reciprocal hit pairs were averaged to eliminate the scoring bias between queries and subjects. To eliminate the impact resulting from genome length differences of these microviral genomes, bit scores of hsps were summed for each query–subject pair and normalized by the total length of two genomes. Normalized bit scores of query–subject pairs were used as the weight of edges to build the binary profile of genomic relationship network. The layout of the network was calculated from the binary profile based on the Force Atlas algorithm. The modularization of the intergenomic relationship network was calculated with the default resolution. The gene calling of vB_VpaM_PG19 and 151 vB_VpaM_PG19-associated genomes was performed using Prodigal (48) with the mode of “meta” and forcing the full motif scan. The ends of genomes were closed by parameter “-c,” so no ORFs were produced crossing the ends. The predicted ORFs were subjected to Diamond performing all-to-all BLASTp. The results were screened using RBB (reciprocal best-BLAST) criteria, resulting in 10,713 orthologous pairs. All microviral orthologous pairs were subjected to OrthoMCL (2.0.9) grouping 407 protein clusters (PCs) and singletons (SGs). The species-to-PCs value matrix was built in the order of modules generated by Gephi, and calculated clustering heatmap of PCs with “complete” algorithm. As all the reference sequences labeled *Microviridae* in the NCBI virus database were larger than 4,000 bp in length, sequences with genome length less than 4,000 bp were deleted from the heatmap. The proposed viral clusters (VCs) were identified in accordance with the specific presence or absence of some PCs in modules, which were manually corrected to determine the distinguishing criteria and boundaries among different proposed VCs.

### Ethics statement.

This article does not contain any studies with animals or human participants performed by any of the authors.

### Data availability.

The genome sequence of vibriophage vB_VpaM_PG19 has been deposited in GenBank under accession number OM055665. The 16S rRNA sequence of the host also has been deposited in NCBI under accession number GU460378.1.
