# Supplementary material for: Characterization and Genomic Analysis of ssDNA Vibriophage vB_VpaM_PG19 within Microviridae, Representing a Novel Viral Genus
Source: Microbiol Spectr. 2022 Jul 6;10(4):e00585-22. doi: 10.1128/spectrum.00585-22 (PMC9431446; doi:10.1128/spectrum.00585-22)
Supplement: Supplemental file 1 — Supplemental material. Download spectrum.00585-22-s0001.pdf, PDF file, 0.1 MB [file spectrum.00585-22-s0001.pdf]

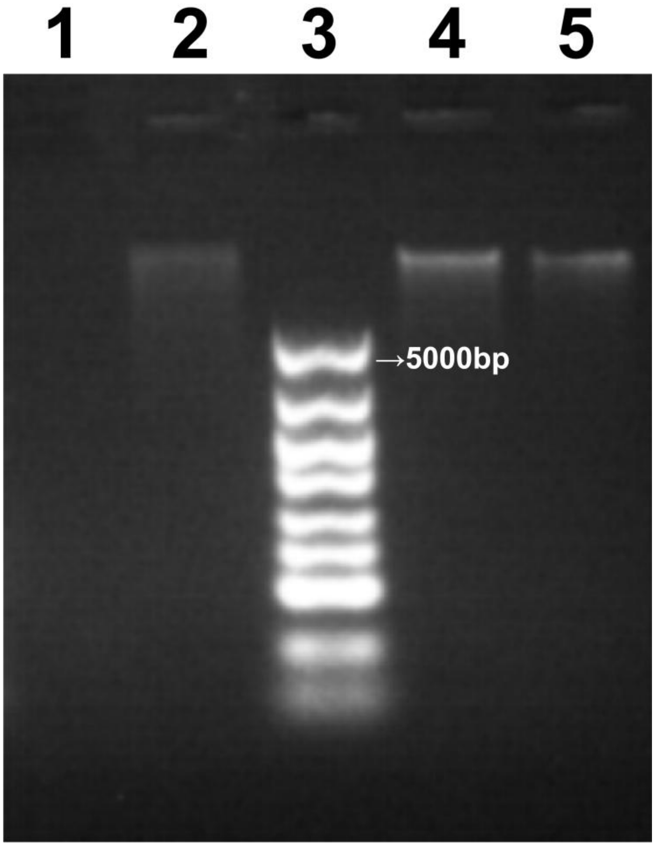

Fig. S1 Agarose gel electrophoresis analysis of the genomic DNA of vibriophage vB_VpaM_PG19. The phage nucleic acids were treated with DNase I (1), S1 nuclease (2) and RNase A (4) at 37°C for 1 h before they were loaded on the agarose gel. Lane 3 represents a 100-5000bp dsDNA marker and lane 5 represents the genomic DNA without digestion.
